# The South African community pharmacy sector—an untapped reservoir for delivering HIV services

**DOI:** 10.3389/frph.2023.1173576

**Published:** 2023-07-14

**Authors:** Tsitsi Nyamuzihwa, Angela Tembo, Natalie Martyn, Francois Venter, Jacqueline Maimin, Juliet Houghton, Samanta Tresha Lalla-Edward

**Affiliations:** ^1^ Ezintsha, Faculty of Health Sciences, University of the Witwatersrand, Johannesburg, South Africa; ^2^ Independent Community Pharmacy Association, Kenilworth, South Africa; ^3^ Southern African HIV Clinician Society, Houghton, South Africa

**Keywords:** antiretroviral therapy, pre-exposure prophylaxis, post exposure prophylaxis, PIMART, primary care drug therapy

## Abstract

Differentiated service delivery is recommended to improve the uptake of HIV testing and treatment for people living with HIV. One service delivery option yet to be fully capitalised on is community pharmacies. There are approximately 3,580 registered community pharmacies in South Africa. A total of 1,110 (31%) of these pharmacies are corporate chain pharmacies located in cities and towns, the remainder are individually owned, many of which are in less populated poorer settings. Community pharmacies traditionally play a pivotal role in providing health education to the populations they serve and are the first point of contact for people seeking health services, offering more convenient opening hours and shorter waiting times than public sector clinics or private doctors. As a result, patients regularly seek a variety of sexual and reproductive health services at community pharmacies such as self-testing devices for HIV, treatment for sexually transmitted diseases, and an array of reproductive health services, spanning emergency contraception to fertility advice, often signifying HIV risk. This has presented an opportunity for community pharmacies to provide access to HIV prevention and treatment to ensure the targets for HIV services set by international agencies and local government are achieved. Despite obstacles experienced with the expansion of the community pharmacist's role, exploring the potential of pharmacies to mediate the existing challenges with HIV service delivery has emerged as an important resource. Assessing the South African communities' specific HIV treatment needs and willingness to access HIV services from community pharmacies will benefit from additional research.

## 1. Introduction

Although there has been international commitment to achieving the Joint United Nations Programme on HIV/AIDS (UNAIDS) goals, considerable work needs to be done to ensure the 95-95-95 targets for HIV services are met by the year 2025. By the end of 2021, 85% of people living with HIV (PLHIV) knew their status, of these, 75% were accessing antiretroviral therapy (ART), and 68% of PLHIV were virally suppressed (1). In sub Saharan countries generally, there are various reasons (socio-economic, contextual and behavioural) for the lag in reaching the 2025 targets (2). South Africa in particular has the largest HIV programme in the world with an estimated 74% of PLHIV on ART; however, more than 2 million South Africans who are HIV positive are not on ART (1). Despite the significant gains South Africa has made in providing access to HIV testing and treatment, gender inequality in accessing HIV services, geographical factors, and socio-economic factors could delay the achievement of the 95-95-95 targets by 2025 (3). To galvanise engagement in HIV testing and treatment as well as to reach underserved and hard to reach populations, the authentic experience of HIV susceptible populations and PLHIV needs to be addressed (4). One way of doing this is through differentiated service delivery (DSD).

The World Health Organization advocates DSD to support the cascade of care for PLHIV as well as those who are vulnerable to infection (5). This approach envisions optimising HIV care particularly for patients in low income countries as well as providing a more individualised service to HIV patients (6), in a manner that minimises strain on health systems. In the *Decision Framework for Antiretroviral Therapy Delivery*, the out-of-facility individual model promotes the use of spaces beyond the traditional hospital or clinic facilities. One such alternative space is the community pharmacy (7).

Globally, community pharmacy operations and offerings differ. In South Africa, community pharmacies retail both prescription and over-the-counter medication in addition to providing health advice. Ilardo ML and Speciale A (2020) explain that community pharmacists’ services are “relatively under-utilised” and this is largely due to the public perception of them as retailers and their exclusion in healthcare policies. Notwithstanding these factors that undermine the profession, the community pharmacist apart from dispensing medicine can also serve as an educator and facilitate treatment adherence (8). A recent systematic review of studies conducted across the world, indicated that interventions driven by community pharmacists have indeed led to a boost in adherence and patients' ability to manage chronic conditions such as hypertension and asthma (9). Another study which surveyed health professionals in rural Australia also concluded that a large majority (73%) of health professionals advocated for expanded healthcare services to be delivered through community pharmacies. Upon comparing professional groups in this study, doctors were only half as supportive, citing fears that community pharmacists lacked the specific expertise required to provide accurate diagnoses and medical advice, although no evidence was supplied to validate their concerns (10).

Community pharmacies often provide extended services and convenience, when compared to other health facility offerings, especially primary care clinics, with many countries reporting growing patient acceptance of community pharmacies' role in providing care (11). The increasing acceptability of community pharmacies is critical in the South African context, where, despite the advances that South Africa has made in the identification and treatment of HIV, further attainment of treatment and prevention targets are hampered because over-burdened primary clinic facilities are unable to meet patients' healthcare needs (12). Community pharmacies in South Africa can provide routine HIV testing and treatment services to alleviate some of the public health facilities' constraints and service delivery barriers such as extended waiting times, high patient to healthcare worker ratios, and the dependence on healthcare personnel in clinics and hospitals (13). Furthermore, community pharmacies are not stigmatised and are frequented by many of the harder to reach population groups such as men and young women (14, 15).

## 2. Proliferation of community pharmacies

The ownership of community pharmacies in South Africa was historically restricted to registered pharmacists. However, in 2003 regulations opened the ownership of community pharmacies to lay persons under the direct supervision of a responsible pharmacist, fuelling the emergence of corporate community pharmacy chains, and subsequent expansion in the number of pharmacies (16–18).

**Figure 1:**
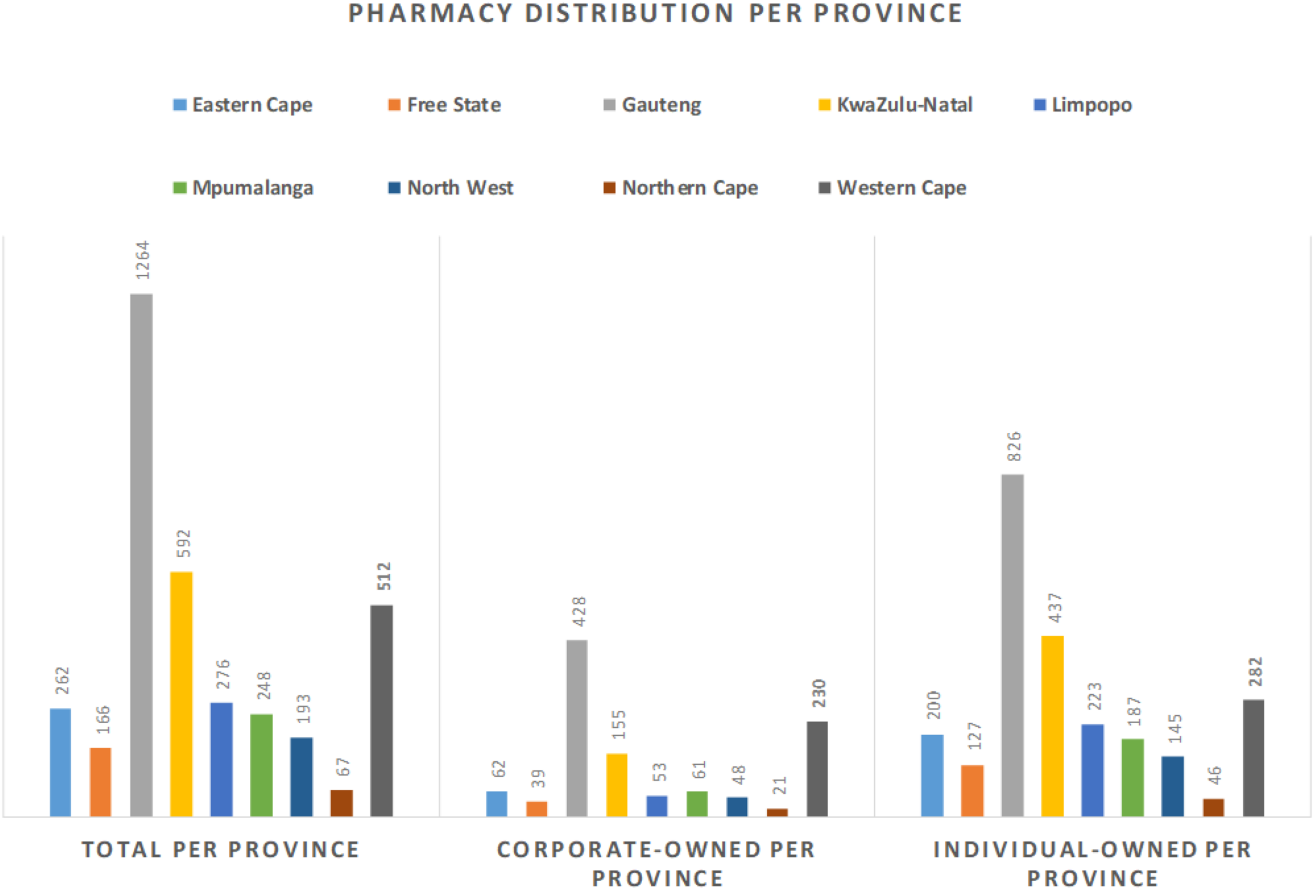
Pharmacy distribution per province in South Africa.

According to the 2021 South African Pharmacy Council (SAPC) records, approximately 3,580 community pharmacies are registered in South Africa. These pharmacies have multiple urban and rural locations. The geographical distribution of these community pharmacies is aligned with population occupancy and economic contribution—in other words, there is a greater number of pharmacies in more populous areas and in those which have more robust economies (19–21). This implies that rural areas which are generally poorer and have smaller populations have not seen the same expansion in pharmacies in comparison to urban settings. This trend is also identified by Moodley and Suleman (18), and further illustrated in Figure 1, where the total number of community pharmacies is highest in Gauteng, the most populous province (22) and lowest in Northern Cape, the province with the smallest population (22).

Approximately 1,253 (35%) of the registered community pharmacies are located in Gauteng Province. Furthermore, around 1,110 (31%) of community pharmacies are corporate pharmacies and the balance are independently owned. The proportion of corporate versus independent community pharmacies is highest in Gauteng (40% vs. 33%) and Western Cape (21% vs. 11%). The converse is true for the other seven provinces which have a higher proportion of independently owned community pharmacies. An estimated 40% of independent community pharmacies are in rural underserviced communities (23).

The number of community-based pharmacies is steadily increasing in South Africa. In 2020 and 2021 alone 648 new community pharmacies were opened in South Africa and the possibility of increasing healthcare services to the wider population via these pharmacies looks promising. The major corporate chain pharmacies in South Africa currently are Clicks and Dis-Chem pharmacies with 618 and 183 pharmacies respectively nationwide (24, 25). Due to their huge revenues, both Dis-Chem (operating profit of ZAR1.5 billion in 2022) and Clicks (operating profit of ZAR3.3 billion in 2022), have further growth strategies to increase their networks countrywide.

Notwithstanding their expansion, one drawback particularly for the growth and sustainability of independent pharmacies is the lack of uniformity regarding co-payment amounts for medically insured customers (26). To prevent the collusion of corporate pharmacies and medical aids in implementing designated service providers (DSPs) which resulted in patients paying heavy co-payments at pharmacies outside the DSPs, the Council for Medical Schemes published regulations which declared these business practices undesirable (27). While, initially, this has tremendously assisted independent pharmacies to continue operating in underserviced areas, the regulations are being held in abeyance until the matter is heard by the appeal committee (23).

## 3. Expanding the role of the community pharmacist

Gray et al., 2019 claim that the pharmaceutical profession is evolving from primarily dispensing and compounding to a more patient orientated primary healthcare provider role (17). Pharmacists and pharmacy based nurses already perform immunizations and screen for a substantial number of conditions including but not limited to hypertension, diabetes, HIV and cholesterol (15). A survey of a group of South African pharmacists showed that while pharmacists were willing to expand their professional services, this extension of services would need to be accompanied by upskilling (28).

There are some pathways available to pharmacists wishing to upskill and expand their professional services. As per the regulations relating to the scope of practise of pharmacy (29) pharmacists can complete primary care drug therapy (PCDT) supplementary training offered by several tertiary institutions. The training which was first introduced as early as the 1990's, aims to equip pharmacists with the necessary skills for comprehensive patient management at primary care level. Upon successful completion of the training, a PCDT pharmacist is granted a Section 22A(15) permit by the Director General of Health which will authorise the pharmacist to diagnose, prescribe and administer medicine for selected conditions in line with primary healthcare level standard treatment guidelines and the essential medicine list. Such conditions include management of chronic conditions such as hypertension and diabetes as well as prescription of antiretroviral post exposure prophylaxis (PEP) to health workers. Currently this list does not include the management of ART.

In 2018, pharmacy interest groups, representing the independent pharmacy groups and the corporates, approached the Southern African HIV Clinicians Society (SAHCS), a large well-respected professional group responsible for local treatment guidelines and advocacy, for guidance on how they could support the prescription of antiretroviral medication through pharmacies. SAHCS subsequently developed a Pharmacy Initiated Management of Antiretroviral Therapy (PIMART) short course for pharmacists and pharmacy-based nurses with the guidance of infectious diseases specialists. The PIMART course which was approved by the SAPC in June 2020, allows appropriately trained pharmacists and nurses working within their pharmacies, to prescribe antiretrovirals as pre-exposure and post exposure prophylaxis, and for treatment, according to a set of adapted Department of Health guidelines, with clear referral pathways. Pharmacists and pharmacy based nurses who have completed the course are currently awaiting approval of Section 22A(15) permits by the Director General of Health which will authorise them to initiate and manage patients on ART. The expansion of the role of the community pharmacist in providing access to ART could see an improvement in HIV testing, prevention and treatment in South Africa.

## 4. Community pharmacies as a potential platform for HIV products and services

Pharmacies in low- and middle-income countries are often the first point of contact with the healthcare system and the most consulted health-care providers (30–33). The increased number of community pharmacies bodes well for South Africa as most of these pharmacies are located in areas easily accessible by the general public such as transport hubs and community shopping centres, potentially increasing access points for HIV service delivery. While there remains an unequal distribution of community pharmacies between rural and urban areas, they remain a very important potential option for providing HIV and sexual health interventions particularly to under serviced communities that have limited access to healthcare.

In the face of health-sector shortcomings and the impact of unanticipated disasters like the COVID-19 pandemic which disrupted public health services such as HIV and tuberculosis testing and reproductive health services (34), exploring the potential of pharmacies to mediate the existing challenges has emerged as an important resource. Data analysed from the public sector indicated a 22% decline in HIV testing, a 26% decline in tuberculosis Gene-Expert tests conducted and a 6% decline in contraceptives prescribed for the period March 2020 to December 2020 (35). The gap in healthcare access was filled in part by people accessing services from pharmacies (34).

In a recent review conducted to evaluate the potential of pharmacy-delivered HIV services in sub-Saharan Africa, there was in increase in uptake of HIV self-tests and in some instances, particularly where people engaged in risky sexual behaviour pre-exposure prophylaxis (PrEP) was sought out more at private pharmacies than at clinics (15). Whilst the reviewers acknowledged that this was early evidence it did suggest that pharmacy-based HIV service delivery models were possible and largely accepted by clients without compromising clinical outcomes (15). Furthermore, during the COVID-19 pandemic, in some Eastern Mediterranean Region countries where clinics were closed, pharmacists played a pivotal role in providing guidance on the management of sexual and reproductive health issues (36).

Assessing the South African communities' specific HIV treatment needs and willingness to access their HIV medication from community pharmacies will benefit from additional research. This was demonstrated by Zhu et al., who in their survey driven investigation found that while patients felt comfortable about pharmacists prescribing PrEP, there were also barriers to be overcome before this practice could be fully rolled out (37). A similar conclusion was also reached in another study which indicated that whilst accessing sexual and reproductive health services at pharmacies was viable especially for at risk groups, integrating sexual and reproductive health services into the traditional pharmacy workflow and policy concerns were other obstacles that needed to be resolved (38).

## 5. Conclusion

Community pharmacies present a unique opportunity to help South Africa alleviate the burden on public health systems as well as to overcome the challenges in HIV testing and treatment in South Africa. Given the development of community pharmacies across South Africa, these facilities can increase access to HIV treatment particularly in vulnerable and hard to reach groups. In so doing, South Africa could stand a better chance of achieving its 95-95-95 goals as envisioned by UNAIDS.

## Data Availability

The original contributions presented in the study are included in the article/Supplementary Material, further inquiries can be directed to the corresponding author.
